# Clinical Outcomes after Percutaneous Coronary Intervention for Cardiogenic Shock Secondary to Total Occlusive Unprotected Left Main Coronary Artery Lesion-Related Acute Myocardial Infarction

**DOI:** 10.3390/jcm12041311

**Published:** 2023-02-07

**Authors:** Marcel A. M. Beijk, Julián Palacios-Rubio, Maik J. D. Grundeken, Debbie N. Kalkman, Robbert J. De Winter

**Affiliations:** 1 Department of Cardiology, Amsterdam University Medical Center, University of Amsterdam, Meibergdreef 9, 1105 AZ Amsterdam, The Netherlands; 2 Cardiology Department, Hospital Universitario Son Espases, Health Research Institute of the Balearic Islands (IdISBa), 07120 Palma, Spain

**Keywords:** acute myocardial infarction, cardiogenic shock, percutaneous coronary intervention, mortality, left main coronary artery, total occlusion

## Abstract

Background: Acute myocardial infarction (AMI) with occlusion of an unprotected left main coronary artery (ULMCA) is a rare condition with a high mortality. The literature on clinical outcomes after percutaneous coronary intervention (PCI) for cardiogenic shock secondary to ULMCA-related AMI is scarce. Methods: In this retrospective analysis, all consecutive patients undergoing PCI for cardiogenic shock secondary to total occlusive ULMCA-related AMI were included between January 1998 and January 2017. The primary endpoint was 30-day mortality. The secondary endpoints were long-term mortality and 30-day and long-term major adverse cardiovascular and cerebrovascular events. The differences in clinical and procedural variables were assessed. A multivariable model was created to search for independent predictors of survival. Results: Forty-nine patients were included, and the mean age was 62 ± 11 years. The majority of patients suffered cardiac arrest prior or during PCI (51%). Thirty-day mortality was 78%, of which 55% died within 24 h. The median follow-up of patients who survived 30 days (*n* = 11) was 9.9 years (interquartile range 4.7–13.6), and long-term mortality was 84%. Long-term all-cause mortality was independently associated with cardiac arrest prior or during PCI (hazard ratio [HR] 2.02, 95% confidence interval 1.02–4.01, *p* = 0.043). Patients who survived to the 30-day follow-up with severe left ventricular dysfunction had a significantly higher risk of mortality compared to patients with moderate to mild dysfunction (*p* = 0.007). Conclusions: Cardiogenic shock secondary to total occlusive ULMCA-related AMI carries a very high 30-day all-cause mortality. Thirty-day survivors with a severe left ventricular dysfunction have a poor long-term prognosis.

## 1. Introduction

Cardiogenic shock secondary to an unprotected left main coronary artery (ULMCA)-related acute myocardial infarction (AMI) is a rare disease with a high risk of mortality due to acute left ventricular (LV) failure and malignant tachyarrhythmias [1]. Presumably a significant amount of patients are deceased before being able to contact emergency medical care, and probably only a minority of patients may reach the hospital. An ULMCA as the culprit lesion in the setting of AMI accounts for about 0.6 to 4% of all primary percutaneous coronary interventions (PCI) [1,2,3,4,5,6]. Although several registries have reported characteristics and outcomes of primary PCI involving ULMCA in the AMI setting [2,3,7,8,9,10], only a few have reported outcomes of patients in cardiogenic shock due to total occlusive ULMCA disease [1,6,11,12,13]. Furthermore, long-term follow-up data on these patients is scarce. In the present study, we evaluate the 30-day and long-term outcomes after PCI for cardiogenic shock secondary to total occlusive ULMCA lesion-related AMI.

## 2. Methods

### 2.1. Study Design and Population

**Figure 1:**
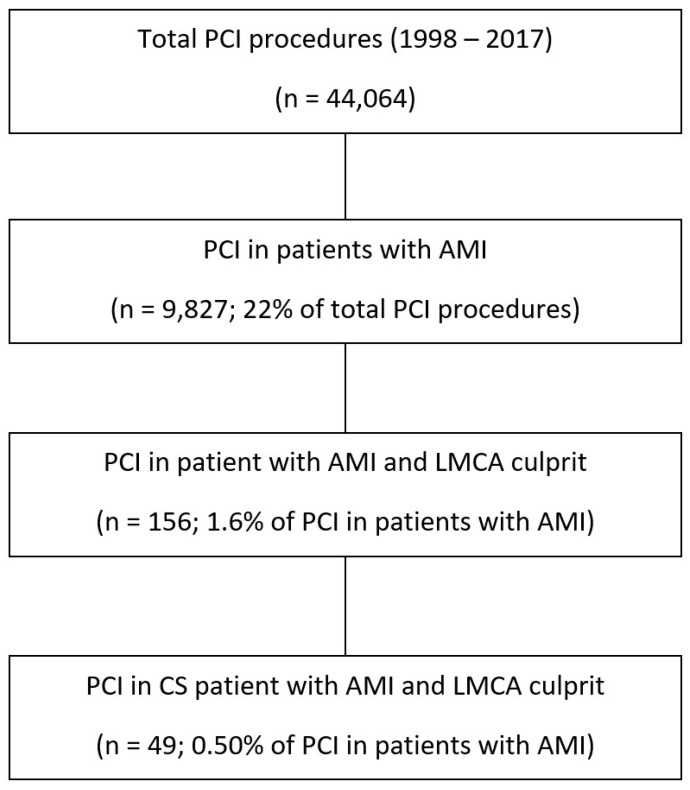
Study flow chart.

This was a retrospective analysis conducted at the Amsterdam University Medical Center, which is a high-volume tertiary referral hospital with 24 h emergency PCI capability and on-site cardiac surgery. Consecutive patients who underwent primary PCI for cardiogenic shock secondary to ULMCA-related AMI between January 1998 and January 2017 were included. Inclusion criteria were consecutive patients aged 18 years or older with an AMI due to culprit coronary lesion in total occlusive ULMCA who presented with cardiogenic shock. Patients with TIMI flow ≥ 1 in the LMCA assessed using angiography were excluded as those patients have a better prognosis [8]. No other exclusion criteria were applied. A total of 49 patients were finally included (Figure 1).

### 2.2. Data Collection and Definitions

The data analyzed in this study was obtained from our prospective institutional database collected at the time of the procedures, including all patients who underwent a PCI. Data on clinical condition at hospital admission and procedural characteristics were screened for the present study. Baseline clinical, angiographic and procedural data, as well as variables related to clinical management, were retrieved for all selected patients. Follow-up data were completed with information obtained from discharge letters and in- and outpatient charts from our hospital or referring centers. Finally, vital status was verified from our hospital database by telephone contact with the patient or the general practitioner in November 2022.

AMI was defined as an acute presentation of prolonged (>30 min) chest pain with (a) electrocardiogram (ECG) changes indicative of myocardial ischemia (persistent elevation of more than 1 mm of the ST segment in two or more contiguous leads on the ECG), or (b) ECG suggestive of myocardial ischemia (i.e., new left bundle branch block or significant ST depression in multiple leads) at the discretion of the treating cardiologist. Cardiogenic shock was defined as (a) systolic blood pressure < 90 mmHg for at least 30 min or the need for supportive measures to maintain a systolic blood pressure > 90 mmHg and (b) signs of end-organ hypoperfusion (altered mental status or confusion, peripheral coldness, oliguria < 0.5 mL/kg/h and blood lactate > 2 mmol/L) [14]. ULMCA was defined as unprotected when there was no coronary artery bypass graft on left anterior descending artery (LAD) and/or ramus circumflex artery (RCx). ULMCA was defined as the infarct-related coronary artery (“culprit lesion”) based on angiography with total occlusion of the left main stem (TIMI 0 flow) due to thrombus. All angiograms were reviewed by an interventional cardiologist (MB) to verify the aforementioned condition. Successful reperfusion was defined as <30% residual stenosis at the lesion site after stenting or <50% residual stenosis after balloon angioplasty and final TIMI 3 flow.

PCI was performed, and adjunctive pharmacological treatment was administered according to standard guidelines at the time [15,16,17]. The use of antithrombotic medications, predilatation, thrombus aspiration devices, intra-aortic balloon pump (IABP) or a left ventricular assist device (LVAD), such as Impella^®^, was at the discretion of the operator. After coronary stenting, dual antiplatelet therapy was prescribed.

### 2.3. Clinical Outcomes

The primary outcome for this analysis was 30-day mortality. Secondary outcomes were long-term mortality, 30-day and long-term major adverse cardiovascular and cerebrovascular events (MACCE) defined as the composite of all-cause mortality, recurrent myocardial infarction (MI), repeat revascularization and stroke. Recurrent MI was assessed retrospectively according to the fourth universal definition of MI [18]. Repeat revascularization was defined as any revascularization of the target coronary artery after the index event, either percutaneous or surgical. Stroke was defined as any cerebrovascular event, either hemorrhagic or ischemic. In the patients who survived to 30-day follow-up, LV ejection fraction (LV EF) was reported as secondary outcome.

### 2.4. Statistical Analysis

The categorical variables are presented as *n* and percentages. The normality of the continuous variables was evaluated using the Shapiro–Wilk test and expressed, accordingly to their distribution, as mean ± standard deviation or median and interquartile range (IQR). Descriptive statistics were used to present the baseline clinical, angiographic and procedural characteristics. Follow-up was censored at the date of last contact. Cumulative event rates were reported at 30 days and long-term follow-up. Differences in clinical and procedural variables were explored, using chi-squared, Fisher’s exact, Student’s *t*- or Mann–Whitney’s U tests as appropriate. When statistically significant or borderline differences (*p* < 0.10) were found, those variables were entered into a Cox proportional hazards regression model. *p*-values provided are two-tailed. All analyses were performed using IBM SPSS Statistics, version 27.0 (Chicago, IL, USA).

### 2.5. Ethics

This retrospective analysis was performed in accordance with ethical principles consistent with the Declaration of Helsinki. Ethical approval for this study was waived by the Medical Ethics Review Committee of the Amsterdam University Medical Center because there were no alterations to routine clinical care.

## 3. Results

A total of 49 patients met the requirements for the analysis. Baseline clinical characteristics are summarized in Table 1. The mean age was 62 ± 12 years, and 42 (86%) patients were male. Cardiac arrest prior or during PCI occurred in the majority of patients (53%). The median time from symptom onset to PCI was 2.2 h (IQR 1.5–3.7).

Angiographic and procedural characteristics are summarized in Table 2. In the majority of patients, femoral access was used (94%), and the right coronary artery (RCA) was dominant in most cases (84%), although in four cases, the RCA was not visualized. A total of six patients had an occluded ULMCA with one-vessel disease, eight patients had an occluded ULMCA with two-vessel disease, and one patient had an occluded ULMCA with three-vessel disease. Mechanical support device was used in 38 patients (57% IABP and 20% Impella^®^), and eight patients were upgraded from IABP to Impella^®^ to provide more LV support. In one patient, the femoral arteries did not allow implantation of mechanical assist device, and in one case, the patient’s relative refused mechanical support. Finally, one patient received a second upgrade to a Heartmate II^TM^ LVAD. Inotropes were administered in 94% of the patients. The patients who did not receive inotropes had a systolic blood pressure <90 mmHg, were not resuscitated prior or during PCI and received an IABP prior to PCI, and the angiogram showed dominance of the right coronary artery with collaterals to the LCA. In 10 (20%) patients, only balloon angioplasty was performed as the majority of these patients were in ongoing cardiogenic shock (with or without restoration of coronary flow), and the operator did not continue with the stent placement. In one case, after balloon angioplasty was performed, the patient was discussed with the cardiac surgeon for emergency coronary artery bypass grafting (CABG), but after implantation of an IABP, the patient arrested and died in the catheterization laboratory. Of all patients, TIMI 3 flow was achieved in 26 (53%) patients, and in 10 (20%) cases, there was no reperfusion (TIMI 0/1) post PCI.

### Clinical Outcomes

Thirty-day and long-term clinical outcomes were obtained in all patients and are summarized in Table 3. At 30 days, all-cause mortality (primary outcome) was 78% (38 patients), of whom 55% (27 patients) died within the first 24 h after admission. At the 30-day follow-up, MACCE occurred in 82% of patients. Two patients underwent repeat revascularization of the LM, one patient by PCI the next day to optimize the stent result, and one patient was treated with balloon angioplasty only, followed by emergency CABG due to three-vessel disease. One patient underwent staged PCI of the RCA (non-target vessel) within 1 month. At the 30-day follow-up, one patient suffered a stroke and died 3 days later.

**Figure 2:**
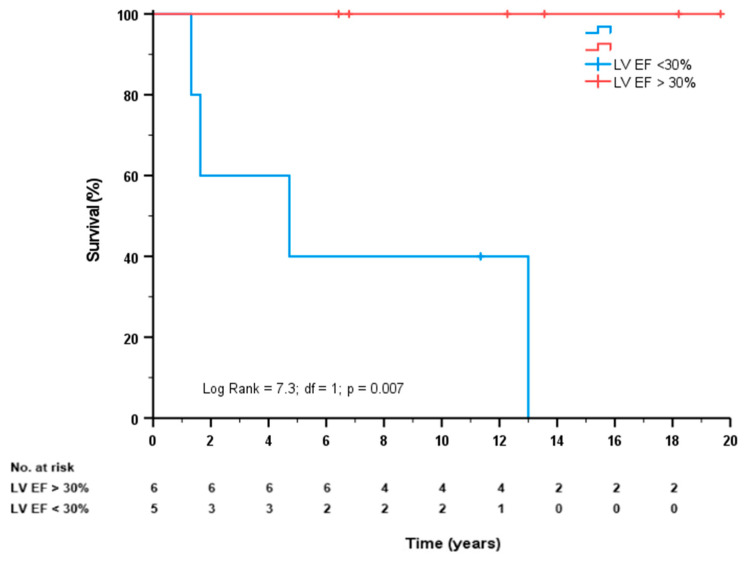
Survival curves for patients with mild to moderate LV dysfunction (LV EF > 30%) (red line) or severe LV dysfunction (LV EF < 30%) (blue line).

Long-term follow-up was available in 11 patients (alive after 30 days), and the mean follow-up duration was 9.9 years (IQR 4.7–13.6) years. Of these patients, five (46%) had severe LV dysfunction and received an implantable cardioverter defibrillator (ICD), while three patients had mild LV dysfunction, and three patients had moderate LV dysfunction. The assessment of LV dysfunction was performed using echocardiography (*n* = 5), nuclear scintigraphy (*n* = 5) and cardiac magnetic resonance (*n* = 1). During long-term follow-up, an additional four patients died, who all had severe LV dysfunction. Figure 2 shows the survival curves for patients with LV EF > 30% or LV EF < 30% (log rank = 7.3; df = 1; *p* = 0.007). The MACCE at long-term follow-up was 86%, as during follow-up, three patients suffered a myocardial infarction, and one patient suffered a stroke. No additional revascularization was performed during follow-up.

**Figure 3:**
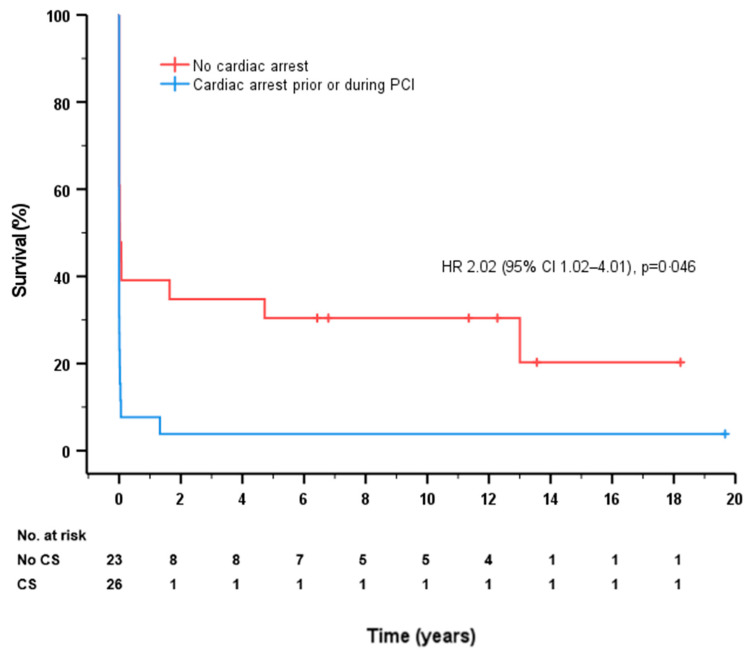
Survival curves for patients without cardiac (red line) or cardiac arrest prior or during PCI (blue line).

The survival differences for every subgroup are shown in Table 4. Thirty-day all-cause mortality was higher in patients with cardiac arrest prior or during PCI (92% vs. 61%, *p* = 0.004) and balloon-only angioplasty (100% vs. 72%, *p* = 0.027) but was lower in patients with successful reperfusion (65% vs. 91%, *p* = 0.026) and mechanical assist device use (74% vs. 91%, *p* = 0.041). The patients who were deceased at 30 days were significantly older (difference of 7.5 years). We found no statistically significant differences in mortality depending on the time to puncture or among other variables. These differences between groups were consistent at the end of follow-up except for age and mechanical support device use. In addition, the long-term all-cause mortality was lower in patients when collaterals arising from the RCA were present (25 vs. 90%, *p* = 0.017). A Cox survival model, including age, smoking, cardiac arrest prior or during PCI, successful reperfusion and mechanical assist device use, did not show predictors for 30-day all-cause mortality. A Cox survival model, including cardiac arrest prior or during PCI, successful reperfusion and mechanical assist device use, showed that long-term all-cause mortality was independently predicted by cardiac arrest prior or during PCI (hazard ratio [HR] 2.02, 95% confidence interval [CI] 1.02–4.01, *p* = 0.043). The survival curves for cardiac arrest prior or during PCI are shown in Figure 3.

## 4. Discussion

The present study features the clinical outcomes of patients who underwent PCI for cardiogenic shock secondary to total occlusive ULMCA lesion-related AMI. To the best of our knowledge, the present study provides the longest follow-up available. The main findings from the present study are as follows: (a) patients who undergo PCI for cardiogenic shock secondary to ULMCA lesion-related AMI with pre-procedural TIMI flow 0 at angiography have a very high risk of mortality at 30 days and long-term follow-up; (b) patients without cardiac arrest prior or during PCI are more likely to survive; and (c) patients with a severe LV dysfunction after 30 days are less likely to survive to long-term follow-up.

The incidence of ULMCA lesion-related AMI has been reported to range from 0.8 to 2.5% in ST-elevation myocardial infarction (STEMI) patients undergoing cardiac catheterization [1,3,4,11,19]. In the present study, the incidence was 0.50%, a discrepancy related to the fact that previous studies also included subtotal occlusion or critical stenosis of the LMCA. The present study exclusively included patients with total occlusive ULMCA AMI referred for primary angioplasty and is in line with the findings of the Acute Left Main Coronary Total Occlusion (ATOLMA) Registry, as they reported an incidence of 0.58% [20]. Presumably, the true incidence of ULMCA lesion-related AMI may be underestimated as patients in this clinical setting may die before they reach hospital.

Coronary perfusion via the LMCA leads to a large area of LV myocardium, and LMCA disease is associated with extensive myocardial ischemia with a high risk of cardiogenic shock and cardiac arrest. Prompt revascularization is paramount to improve clinical outcomes. Even though primary PCI for ULMCA AMI has been performed with increasing frequency over the past decades, it remains a significant challenge, and long-term follow-up in survivors is limited. Table 5 provides an overview of previous studies including patients with AMI due to ULMCA (electronic search of the MEDLINE and Cochrane Central databases conducted in November 2022, using MeSH terms “left main coronary artery” and “myocardial infarction”). In line with previous studies, we show that all-cause mortality in patients with an ULMCA-related AMI is very high, and it has not changed with time. However, the studies display a remarkable heterogeneity in patient condition on admission and mortality. The inclusion of cardiogenic shock ranges from 12 to 76%, TIMI flow varies from 0 to 3, and 30-day mortality ranges from 6.2 to 78%. These mortality differences might well be explained by the prevalence of cardiogenic shock, and this might be due to a selection bias where longer times to PCI naturally discard sicker patients. Actually, studies where time from symptom-onset to PCI is longer than 4 h reported 13.2% patients in shock, while studies with times shorter than 4 h have 57.8% of patients in shock (*p* < 0.001). In the current study, the median symptom-onset to PCI time was 2.2 h (IQR 1.5–3.7), which is comparable to previously reported intervals of 2.2–3.4 h [1,8,13]. Our data show that most patients die within the first 24 h. All this suggests that many patients with a total occlusive ULMCA are probably too sick to survive despite state-of-the-art medical care as they have sustained severe cardiac and multiorgan damage.

In particular, ULMCA-related AMI with cardiogenic shock is independently associated with mortality [4,27,41]. In a meta-analysis of 13 studies evaluating primary PCI for ULMCAs as culprit lesions in AMI, 26% of patients presented in cardiogenic shock. The average 30-day all-cause mortality was 15% in patients presenting without cardiogenic shock and 55% in patients presenting with shock [8]. The present study focuses on ULMCA-related AMI with cardiogenic shock in patients with TIMI flow 0 at angiography and shows an 30-day all-cause mortality of 78%. Only a few comparable studies have evaluated these patients at highest risk of mortality. The ATOLMA registry [20] is the only study that exclusively included patients with total occlusive (TIMI flow 0) ULMCA-related AMI, but the percentage of cardiogenic shock was “only” 89%. Two other studies exclusively included patients in cardiogenic shock due to ULMCA-related AMI but were liberal in the inclusion of pre-procedural TIMI flow (i.e., 0 to 3) [39,40]. The use of mechanical support was comparable between the studies and the present study, except for the study by Yeoh et al. with a remarkably low percentage of merely 8% [39]. In the abovementioned studies and the present study, the only common predictor significantly associated with reduced 30-day survival was successful reperfusion (final TIMI flow 3). Although in the present study, the use of mechanical support was found to be associated with 30-day mortality, in the studies by Galván-Román et al. [40] and Gutiérrez-Barrios et al. [20], this was not observed. In the study by Galván-Román et al. [40] and the present study, mechanical support was inserted before PCI in the majority of cases. In line, a systematic review and meta-analysis showed that routine use of IABP in STEMI patients complicated by cardiogenic shock is conflicting [42]. This was confirmed in the randomized IABP-SHOCK II trial, including approximately 9% of ULMCA STEMI patients, in whom the use of IABP did not significantly reduce mortality in patients with cardiogenic shock complicating AMI [43]. The study by Galván-Román et al. [40] and the present study also included patients treated with the Impella^®^ LVAD that increases cardiac output without increasing myocardial work, which might be more suited for use in cardiogenic shock. However, in the randomized IMPRESS trial, the Impella^®^ CP was compared with the IABP in patients with cardiogenic shock complicating AMI [44]. Including a minority of patients treated for ULMCA disease, the study showed that routine treatment with Impella^®^ did not affect 30-day mortality. In contrast, in a small nonrandomized study, the 30-day outcome with Impella^®^ 2.5 percutaneous LVAD support initiated either prior (*n* = 20) to or after (*n* = 16) PCI in cardiogenic shock patients with ULMCA culprit lesions in the context of AMI was evaluated [45]. With a significant difference in preprocedural TIMI flow (prior group: TIMI flow 0 or 1 in 18% vs. after group: TIMI flow 0 or 1 in 67%, *p* < 0.001), the 30-day all-cause mortality was 51.9% in the prior group and 87.5% in the after group (*p* = 0.004). In our institution, the Impella^®^ 2.5 became available in 2004 and Impella^®^ CP in 2012. Of the eight patients who were upgraded from IABP to Impella^®^ support, all but one had died at the 30-day follow-up. Despite the frequent use of mechanical support, mortality remains very high in ULMCA AMI patients, especially with a total occlusive left main, and neither IABP nor Impella^®^ may not provide sufficient support in the setting of extensive myocardial stunning. Whether aggressive mechanical support strategy with extracorporeal membrane oxygenation (ECMO) will improve outcomes is still to be investigated. In a small single center, observational study, 17 cases of cardiogenic shock due to ULMCA thrombosis (TIMI flow 0–1) received immediate IABP, followed by PCI of all the diseased lesions and provisional ECMO in the catheterization laboratory [46]. Out of the seven patients treated with ECMO, five (71%) survived to the 1-year follow-up. Currently, in several studies, ECMO or TandemHeart support was used, but the comparison of mortality rates is hampered as the inclusion criteria and prevalence of cardiogenic shock varies strongly [1,13,20,24,29,31,37,40].

Right coronary artery dominance has been suggested by some authors as possible grounds for differences in survival [11,20]. This is explained by the fact that right coronary artery dominance has a greater division of vasculature supplying the left ventricle (into three “parts”), whereas left coronary artery dominance means that most of the myocardium is essentially dependent on two arteries [47]. In patients with a total occlusive ULMCA, this division of vasculature dependent on coronary dominance becomes even more relevant as the LMCA supplies those two arteries. Accordingly, it is not surprising that right coronary artery dominance has been related to survival in patients with subtotal LMCA occlusion [11,20]. In the ATOLMA registry [20], a 100% incidence of right coronary artery dominance was observed, whereas in the present study, 84% of patients had right coronary artery dominance. Of the 30-day survivors (*n* = 11) in this study, all patients did have right coronary artery dominance. Secondly, the presence of collateral circulation may be a predictor of survival, but the results are conflicting [48,49]. In the ATOLMA registry, the RCA was visualized in only 50% of cases; in cardiogenic shock patients, the absence of collateral circulation was significantly higher which may emphasize the importance of these collaterals [20]. In the present study, the RCA was visualized in more than 90%, but only 8.2% (four patients) had collaterals arising from the RCA to the LCA. Of the 30-day survivors in the present study, 3 out of 11 patients had collaterals arising from the RCA. In addition, long-term all-cause mortality was significantly lower in patients with the presence of collaterals arising from the RCA (25 vs. 90%, *p* = 0.017). Finally, as far as we know, the present study is the only one to report on LV function and ICD therapy at follow-up after 30 days. Of the 30-day survivors (*n* = 11), 46% had severe LV dysfunction and received ICD therapy, while six patients had a mild to moderate LV dysfunction. All patients who died during long-term follow-up had documented severe LV dysfunction. In contrast, of the long-term survivors (*n* = 7) all but one patient had mild to moderate LV dysfunction (*p* = 0.007). In three out of the six surviving patients with mild to moderate LV dysfunction, collateral circulation was observed.

Recently, a subanalysis from the CULPRIT-SHOCK trial evaluated radial versus femoral artery access for PCI in patients with AMI and multivessel disease complicated by cardiogenic shock [50]. The radial approach was associated with a lower 30-day rate of death (34.7% vs. 49.7%; OR: 0.56; 95% CI 0.33–0.96) and a lower 30-day rate of renal replacement therapy (5.9% vs. 15.9%; OR: 0.40; 95% CI 0.16–0.97). In contrast, no significant differences were observed regarding the 30-day risks of type 3 or 5 Bleeding Academic Research Consortium bleeding and stroke. The observed reduction in death with transradial access was no longer significant at 1 year (42.4% vs. 55.5%, OR: 0.78; 95% CI 0.46–1.32). In the studies by Galván-Román et al. [40], Gutiérrez-Barrios et al. [20], Yeoh et al. [39] and our study, the transradial access was only performed in 45.7, 34.8, 4.4 and 6%, respectively. Transfemoral access may be preferred when the operator anticipates the use of Impella^®^ or IABP. Further research is needed to evaluate whether early outcomes can be improved by the use of transradial access.

As with any retrospective analysis with a small sample size, this study has limitations. Observational studies, such as the present single-center study, are prone to biases from the non-random assignment of exposures. Secondly, due to the vast time range (nearly 20 years), changes in medical protocols and management occurred. Thirdly, mechanical support was limited to IABP and Impella^®^; none of the patients in our cohort were treated with ECMO.

## 5. Conclusions

Cardiogenic shock secondary to total occlusive ULMCA-related AMI carries a very high 30-day and all-cause mortality. Patients with cardiac arrest prior or during PCI are less likely to survive 30 days. Thirty-day survivors with a severe left ventricular dysfunction have a poor long-term prognosis, whereas 30-day survivors with only mild to moderate LV dysfunction have a good prognosis.

## Figures and Tables

**Table 1:** Baseline clinical characteristics.

	(*n* = 49)
Age (years)	62 ± 11
Male	42 (86%)
BMI (kg/m^2^) *	27 ± 4
Risk factors	
Diabetes	6 (12%)
Requiring oral medication	3 (6.1%)
Requiring insulin	1 (2.0%)
Unknown	2 (4.1%)
Hypertension	5 (10%)
Hypercholesterolemia	4 (8.2%)
Family history of CAD	9 (18%)
Ever smoker	13 (27%)
History of	
Myocardial infarction	9 (18%)
Percutaneous coronary intervention	3 (6.1%)
Presentation	
Cardiac arrest prior of during PCI	26 (53%)
Time from symptom-onset to PCI (hours) **	2.2 (1.50–3.74)

Values are *n* (%), mean ± SD or median (1st–3rd quartile). Data missing for * = 16 and ** = 9 cases. BMI: body mass index; CAD: coronary artery disease; PCI: percutaneous coronary intervention.

**Table 2:** Angiographic and Procedural Characteristics.

	(*n* = 49)	
Femoral access	46	(94%)
*Angiographic characteristics*		
Coronary artery dominance		
RCA	41	(84%)
LCA	1	(2.0%)
Balanced	3	(6.1%)
Unknown (RCA not visualized)	4	(8.2%)
Collaterals arising from the RCA	4	(8.2%)
*Procedural characteristics*		
Mechanical support device	38	(78%)
IABP	28	(57%)
LVAD (Impella^®^)	10	(20%)
Upgrade IABP to LVAD (Impella^®^)	8	(16%)
Timing of mechanical support (*n* = 39)		
Immediately before PCI	21	(55%)
Directly after PCI	17	(45%)
Thrombus aspiration performed	9	(18%)
Concomitant medication		
GP IIb/IIIa inhibitors	16	(33%)
Inotropics	46	(94%)
PCI type		
Balloon angioplasty only	10	(20%)
Stent placement	39	(80%)
Stent type (*n* = 39)		
Drug-eluting stent	16	(41%)
Bare metal stent	22	(56%)
EPC capturing stent	1	(2.3%)
Mean stent length (mm)	17.7 ± 5.6	
Mean stent diameter (mm)	3.6 ± 0.4	
No. of stents: (*n* = 39)		
No. of patients treated with one stent	34	(87%)
No. of patients treated with two stents	5	(13%)
TIMI flow after intervention		
TIMI 0	9	(18%)
TIMI 1	1	(2.0%)
TIMI 2	13	(27%)
TIMI 3	26	(53%)

Values are *n* (%), mean ±SD. RCA: right coronary artery; LCA: left coronary artery; IABP: intro-aortic balloon pump; LVAD: left ventricular assist device; PCI: percutaneous coronary intervention; EPC: endothelial progenitor cell; TIMI: thrombolysis in myocardial infarction.

**Table 3:** Outcomes.

	(*n* = 49)	
*Primary outcome*		
All-cause mortality at 30 days	38	(78%)
*Secondary endpoints at 30 days*		
*MACCE*	40	(82%)
AMI	0	(0%)
Revascularization		
Staged PCI	1	(2.0%)
Staged CABG	1	(2.0%)
TLR	2	(4.1%)
Stroke	1	(2.0%)
*Secondary outcomes at long-term follow-up*		
All-cause mortality	42	(86%)
MACCE	46	(94%)
AMI	3	(6.1%)
Revascularization		
Any PCI	2	(4.1%)
Any CABG	1	(2.0%)
TLR	2	(4.1%)
Stroke	2	(4.1%)

Values are *n* (%) or median (1st–3rd quartile). MACCE: major adverse cardiac and cerebrovascular events (defined as the composite of all-cause mortality, AMI, stroke and any revascularization); AMI: acute myocardial infarction; PCI: percutaneous coronary intervention; CABG: coronary artery bypass graft surgery; TLR: target lesion revascularization.

**Table 4:** Univariate analysis.

	Survivors			
	At 30 Days	*p*-Value	Long-Term *	*p*-Value
	(*n* = 11, 22%)		(*n* = 7, 16%)	
*Clinical variables*				
Gender (male vs. female)	9/42 vs. 2/7	0.41	5/42 vs. 2/7	0.25
**Age** (years, alive vs. dead)	56.0 vs. 63.5	0.026	57.9 vs. 62.5	0.16
BMI (kg/m^2^, alive vs. dead) **	27.6 vs. 26.5	0.24	28.2 vs. 26.5	0.17
**Smoking**	5/13 vs. 6/36	0.084	3/13 vs. 4/36	0.13
Diabetes mellitus	1/6 vs. 10/43	0.37	0/6 vs. 7/43	0.14
Hypertension	1/5 vs. 10/44	0.85	0/5 vs. 7/44	0.44
Dyslipidemia	1/4 vs. 10/45	0.97	0/4 vs. 7/45	0.55
Family history of CAD	2/9 vs. 9/40	0.91	1/9 vs. 6/40	0.86
Previous AMI	2/9 vs. 9/40	0.99	1/9 vs. 6/40	0.77
**Cardiac arrest prior or during PCI**	2/26 vs. 9/23	0.004	1/26 vs. 6/23	0.003
Time to puncture *** (hours, alive vs. dead)	4.4 vs. 3.0	0.16	4.3 vs. 3.1	0.24
*Procedural characteristics*				
Vascular access (femoral vs. radial/brachial)	10/46 vs. 1/3	0.56	6/46 vs. 1/3	0.45
Presence of collaterals arising from the RCA ****	3/4 vs. 8/41	0.059	3/4 vs. 4/41	0.017
**Successful reperfusion**	9/26 vs. 2/23	0.026	6/26 vs. 1/23	0.031
**Mechanical assist device used**	10/38 vs. 1/11	0.041	6/38 vs. 1/11	0.073
Thrombus aspiration performed	3/9 vs. 8/40	0.29	1/9 vs. 6/40	0.58
**Balloon only angioplasty**	0/10 vs. 11/39	0.027	0/10 vs. 7/39	0.027
Stent type (BMS vs. DES)	6/22 vs. 5/16	0.89	4/22 vs. 3/16	0.83

Differences in survival for every variable at each time cut-off, comparing survivors (numerator) among patients with the condition (denominator) versus patients without it. Bold letters denote variables for which there are significant/borderline differences. *: patients who survived to 30-day follow-up. **: Data about BMI were missing for 16 cases. ***: Time from symptom onset to procedure was missing for 9 cases. ****: visualization of RCA missing in 4 cases. CAD: coronary artery disease; AMI: acute myocardial infarction; BMS: bare metal stent; DES: drug eluting stent.

**Table 5:** Previous studies on acute myocardial infarction due to unprotected left main coronary stenosis.

First Author (Ref.#)	Study Period	Sample Size	Study Setting	Follow-Up Period (Mean)	Symptom-Onset to PCI	Treatment	Presenting with Shock	Mechanical Support	30-Day Mortality	Long Term Mortality
Izumikawa et al. [13]	1988–2009	72	Two centers (TIMI flow 0–3)	1.7 ± 2.9 years	2.6 ± 0.7 h	15% BA, 69% BMS, 15% DES	46%	89% IABP, 32% ECLS	44% (in-hospital)	>50%
Sakai et al. [6]	1990–2001	38	Single center (TIMI flow 0–2)	1 year	NA	74% BMS	74%	100% IABP	55%	58%
Brueren et al. [21]	1990–2001	35	Single center (TIMI flow NA)	2 (0–5.1) years	1.7 ± 2.1 h	71% stenting	NA	57% IABP	NA	41%
Marso et al. [22]	1994–1996	40	Multicenter, international registry (TIMI flow 0–3)	1 year	1 h (0.5–4.5)	58% BA, 42% BMS	92%	87% IABP	55% (in-hospital)	65%
Grundeken et al. [23]	1998–2008	84	Single center (TIMI flow 0–3)	1 year	2.2 h (1.5–3.7)	51% stenting, 35% CABG, 14% BA/suction	55%	84% IABP, 16% Impella	50%	54%
Puricel et al. [24]	1995–2007	65	Single center (TIMI flow 0–3)	1 year	NA	35% BA, 42% BMS, 23% DES	NA	38% IABP, 11% TandemHeart or Impella	33% (in-hospital)	±50%
Hurtado et al. [25]	1999–2007	71	Single center (TIMI flow 0–3)	2.7 (0.1–7.3) years	NA	85% stenting, 47% DES	59%	54% IABP	47% (in-hospital)	75%
Xu et al. [26]	1999–2013	55	Single center (TIMI flow 0–3)	44.6 ± 31.3 months	1.8 h ± 0.6	11, BA, 82% stenting, 7% no intervention	55%	95% IABP	40% (in-hospital	69%
Parma et al. [27]	2000–2010	58	Single center (TIMI flow 0–3)	3 years	43% within 3 h	7% BA, 27 BMS, 74% DES	52%	52% IABP	40%	60%
Liu et al. [28]	2000–2014	372	Two centers (TIMI flow 0–3)	1 year	40% < 6 h	26% thrombolytic therapy, 1% BA, 12 BMS	8% Cardiac arrest	21% IABP	6.2%	8.1%
Gharacholou et al. [29]	2000–2014	40	Three centers (TIMI flow 0–3)	5 years	NA	45% BMS, 55% DES	43%	48% IABP, 10% TandemHeart	33%	34%
Sadowski et al. [30]	2003–2006	643	Nationwide (Poland) (TIMI flow 0–3)	1 year	NA	57% stenting, 32% no intervention, 7% CABG	16%	12% IABP	23%	31%
Yap et al. [31]	2003–2012	67	Multicenter, international registry (TIMI flow 0–3)	In-hospital	NA	12% BA, 31% BMS, 55% DES	66%	81% IABP, 22% ECMO	48% (in-hospital)	NA
Gagnor et al. [32]	2004–2009	38	Single center (TIMI flow 0–3)	504 ± 653 days	1.3 h ± 0.9	3% BA, 37% BMS, 60% DES	74%	95% IABP	42% (in-hospital)	44%
Jensen et al. [33]	2005–2007	71	Multicenter (Denmark) (TIMI flow 0–3)	1.5 years	NA	89% DES	41%	13% IABP	31%	38%
Pappalardo et al. [2]	2005–2008	48	Two centers (TIMI flow 0–3)	1 year	NA	61% DES, 39% BMS	45%	54% IABP	21% (in-hospital)	29%
Baek et al. [34]	2005–2009	61	Multicenter (Korean registry) (TIMI flow 0–3)	1 year	4.6 h ± 9.1	95% DES, 5% BMS	23%	44% IABP	21.3%	23%
Almudarra et al. [35]	2005–2010	784	British Cardiovascular Intervention Society (BCIS) registry (TIMI flow 0–3)	1 year	NA	36% BMS, 60% DES	41%	39% IABP	28%	38%
Pedrazzini et al. [10]	2005–2010	348	76 centers (TIMI flow NA)	NA	4.3 h (1.9–10.6)	78% DES	12%	14%	11% (in-hospital)	NA
Qin et al. [7]	2005–2016	30	Single center (TIMI flow 0–3)	3 ± 2.2 years	6.4 ± 5.5 h	80% DES, 13% BMS, 7% no PTCA	27%	30% IABP	20% (in-hospital)	20%
Ielasi et al. [36]	2006–2012	34	Single center (TIMI flow 0–3)	8.5 ± 6.2 months	1.5 h ± 0.5	28% BMS, 18% DES	65%	62% IABP	24%	24%
Shibata et al. [37]	2006–2017	134	Multicenter registry (TIMI flow 0–2)	In-hospital	NA	13% BA, 31% BMS, 56% DES	69%	96% IABP, 47% VA-ECMO	55% (in-hospital)	NA
Patel et al. [1]	2007–2012	568	117 centers (TIMI flow 0–1)	3 years	3.3 h (2.2–5.6)	Stenting in almost all patients, 2/3 DES use *	58%	53% IABP, 6.1% CPS	42% (in-hospital)	74%
Homorodean et al. [38]	2010–2017	81	Two centers (TIMI flow 0–3)	3 years	6 h	59% BMS, 41 DES	49%	7% IABP	36%	53%
*Studies with inclusion of either only TIMI flow 0 or 100% cardiogenic shock*										
Current study	1998–2017	49	Single center (TIMI flow 0)	9.9 ± 6.2 years	2.2 h (1.5–3.7)	20% BA, 45% BMS, 33% DES	100%	57% IABP, 20% Impella	78%	84%
Gutiérrez-Barrios et al. [20]	2005–2011	46	Two centers (TIMI flow 0)	1 year	1.9 h ± 0.9	30% BA, 22% BMS, 48% DES	89%	44% IABP, 7% VA-ECMO	59%	61%
Yeoh et al. [39]	2005–2013	45	Multicenter (TIMI flow NA)	1 year/up to 9 years	NA	13% BA, 58% BMS, 29% DES	100%	8% IABP	67%	73%/80%
Galván-Román et al. [40]	2012–2022	70	Two centers (TIMI flow 0–3)	In-hospital	3.4 h ± 5.52	14% BA, 8% BMS, 77 DES	100%	69% IABP, 21% Impella, 27% VA-ECMO	54% (in-hospital)	NA

Cohorts and case series reporting under 30 patients were excluded. * Exact numbers are not provided. PCI: percutaneous coronary intervention; NA: not available; BA: balloon angioplasty; BMS: bare metal stent; DES: drug-eluting stent; CABG: coronary artery bypass grafting; IABP: intra-aortic balloon pump; ECLS: extracorporeal life support; CPS: cardiopulmonary support, VA-ECMO veno-arterial extracorporeal membrane oxygenation.

## Data Availability

Data available on request due to restrictions.
